# Chronic Disease Management, Self-Efficacy and Quality of Life

**DOI:** 10.1097/JNR.0000000000000422

**Published:** 2021-01-13

**Authors:** Sally Wai-Chi CHAN

Chronic diseases are the leading cause of death and disability worldwide. Globally, the prevalence rates of chronic diseases are rising rapidly in all regions and pervading all socioeconomic classes (World Health Organization [WHO], 2014). Many people with chronic diseases have poor illness self-management skills which may be related to low self-efficacy and an aversion to participating in self-management activities. It will lead to poor control of their chronic diseases and consequently poor quality of life. Many people also suffered from more than one chronic disease. Multiple chronic conditions have a substantial impact on individuals’ health and are highly associated with declines in functioning, increased risk of mortality, and substantial healthcare costs. The ways that people cope with their chronic conditions and associated hardships can result in either successful self-management of the condition or low self-efficacy and poor patient-reported outcomes (Cheng et al., 2019).

Self-efficacy refers to an individual’s self-perceived ability to act effectively in a variety of situations It is an important factor influences patients’ ability to self-manage symptoms of their chronic diseases. Self-efficacy plays an important role in determining whether self-care actions are initiated, how much effort is exerted, and how long the effort is sustained in the face of obstacles and failures (Bandura, 1997). Patients with high self-efficacy in coping with their chronic diseases reflect a perceived ability to manage challenges related to their diseases and a sense of control over their lives (Zhu et al., 2018).

Nurses play a significant role in caring for people with chronic diseases. Moreover, patient education is an important nursing role in chronic disease management. Patients with chronic diseases require sustained treatment and self-care over extended periods of time. To successfully manage chronic diseases, patients must learn about their illnesses and practice disease self-management. Nurses may use education to help enhance the self-efficacy, coping, and self-management skills of patients. Hopefully, patients will improve or maintain their physical and psychological well-being and have a good quality of life despite their chronic conditions.

Further, an integrated approach targeting all major, common risk factors of chronic diseases such as cardiovascular diseases, diabetes mellitus, cancer, and chronic respiratory diseases is the most cost-effective way to prevent and control these diseases (WHO, 2014). Nurses are in an ideal position to serve as care coordinators / case managers in integrated care programs to integrate primary, secondary, and tertiary prevention, health promotion, and related programs across different sectors and disciplines. Nurses work cooperatively with multidisciplinary healthcare teams and act as patient advocates within these teams.

This issue of *The Journal of Nursing Research* (JNR) presents studies on chronic diseases, self-efficacy, quality of life, and long-term care. I hope the *JNR* will be your choice for publishing your scholarly work. We also welcome your feedback and suggestions.
